# The effect of gestational age on short- and long-term complications following primary esophageal atresia repair

**DOI:** 10.1016/j.bjane.2024.844546

**Published:** 2024-08-03

**Authors:** Mathias Johansen, Samuel Wasserman, Dan Poenaru, Jean Martin Laberge, Sam J. Daniel, Thomas Engelhardt

**Affiliations:** a McGill University, Montreal Children's Hospital, Department of Pediatric Anesthesia, Montreal, Quebec, Canada; b McGill University Health Center, Montreal Children's Hospital, Department of Anesthesia, Montreal, Quebec, Canada; c McGill University Health Center, Montreal Children's Hospital, Harvey E. Beardmore Division of Pediatric Surgery, Montreal, Quebec, Canada; d Montreal Children's Hospital, Division of Pediatric Otolaryngology, Montreal, Quebec, Canada

Dear Editor,

Esophageal Atresia (EA) is a rare congenital anomaly with an incidence of 1.2 to 4.5 per 10,000 live births.^1^ It is frequently associated with comorbidities which pose significant challenges in the perioperative period.^2^ Although notable progress in treatment options and survival has been made during the past decades, patients with esophageal atresia still encounter long-term complications that severely impact their quality of life. The majority relate to gastrointestinal and respiratory problems which result in multiple hospital admissions as well as diagnostic and therapeutic interventions.^2^, ^3^, ^4^ Prematurity, among other factors, may affect overall long-term outcomes following successful esophageal atresia repair.^5^ This current study hence investigated the impact of gestational age on gastroesophageal and respiratory complications during the first 5 years of life.

We conducted a retrospective chart review including all patients who underwent primary surgical repair at Montreal Children's Hospital between 2005 and 2023, using a predefined paper-based Case Report Form (CRF). The study was approved by the hospital research ethics board (MP-CHU-HSJ-08-003). Prematurity was defined as babies born alive before 37 weeks of pregnancy. Outcomes included patient demographics, medical and surgical history, and ongoing clinical care relating to gastrointestinal and respiratory complications (Table 1). All data were transferred into an electronic REDCap database. Differences between preterm and full-term births were examined using independent *t*-tests or Mann-Whitney *U* test for continuous data and Chi-Squared tests or Fischer exact tests for binomial data. We examined the relationship between gestational age and the incidence of gastrointestinal and respiratory complications using the Chi-Square test and logistic regression. Clinically relevant variables were added to the model to adjust for potential confounding.

**Table 1:** Collected information of all patients who underwent esophageal atresia repair at the Montreal Children's Hospital between 2005 and 2023.

**Demographic data**	Sex, date and place of birth, gestational age, birth weight, Apgar score, date of admission to hospital, date of surgery
**Prenatal data**	Intrauterine growth delay
	Date of diagnosis of esophageal and/or tracheoesophageal fistula
	Multiple pregnancy
	In Vitro Fertilization
**Additional anomalies**	Skeletal, anorectal, cardiac, renal, limb, gastrointestinal, respiratory, other
**Type of esophageal atresia**	Gross classification
**Syndromes**	Vacterl, charge, Other
**Gastrointestinal outcomes**	Medications during first 5 years of life: proton pump inhibitors; cisapride; domperidone; H2 receptor blocking agents;
	Need for gavage feeding
	Esophageal pathology (esophagitis, barret's esophagus, Stenosis, Hiatal hernia, esophageal diverticulum);
	Stomach and duodenal pathology (ectopic pancreas, microgastria) and abnormal histology (peptic esophagitis, eosinophilic esophagitis, dilatation required)
**Respiratory outcomes**	Medications during first 5 years of life (antibiotics, per so steroids, inhalational steroids, ventolin)
	Deteriorations; admission to hospital (intensive care unit, emergency, ward);
	Diagnostics (pneumonia, bronchitis, sinusitis, asthma attack); abnormal respiratory symptoms (coughing, morning cough, night-time cough, grumbling breathing, grumbling (eating), wheezing, respond to ventolin); exertional dyspnea; continuous dosing aerosol (corticosteroids, without long-acting beta agonists, With long-acting beta agonists, other inhaler) and abnormal auscultation (rhonchi, crackles, wheezing)

A total of 137 patients (39.4% females with a mean Gestational Age (GA) of 37.2 ± 3.2 weeks and a mean birth weight of 2679 ± 708 g) underwent esophageal atresia repair and were included in this analysis. The anatomical variations stratified according to the EA Gross classification included Type A = 3%, Type B = 1.6%, Type C = 82.2%, Type D = 4.8% and Type E = 8%.^6^ A VACTERL association was observed in 17 patients (27%) and CHARGE syndrome in one patient (1.6%). Congenital anomalies affected vertebral (23%), anorectal (9.5%), cardiac (45.2%), renal (27.9%), skeletal (11.1%), respiratory (8.6%), digestive (54.7%), and other (20.7%) systems. Preterm patients more frequently required gavage feeding (54.5% vs. 14.8%, OR = 6.67 [1.33 to 33.33], *p* = 0.019) and had stenosis (72.7% vs. 37.0%, OR = 4.55 [0.97 to 20], *p* = 0.046), but were prescribed anti-H2 less frequently (18.2% vs. 77.8%, OR = 0.063 [0.011 to 0.38], *p* = 0.001).

The incidence of respiratory complications was higher in the full-term patient population (respiratory deteriorations 57.7% vs. 33.3%, abnormal respiratory symptoms 84.6% vs. 66.7%, and abnormal auscultation 73.1% vs. 46.7%), with the exception of exertional dyspnea which was higher in the preterm population (13.3% vs. 7.7%). None of the results reached statistical significance.

Our demographic study population closely aligns with previous reports. The use of anti-H2 blocker therapy was significantly higher in full-term patients, while the number of cases of gavage feeding, and stenosis was higher in the premature population. These findings suggest that a subgroup of gastrointestinal complications may be related to gestational age. Future prospective studies should explore additional factors and underlying mechanisms. No association between gestational age and respiratory complications during the first 5 years of life was observed. The study has several limitations, including small sample size, missing data, and variability in reporting of outcomes. Study limitations are attributed to the retrospective data collection in a single institution, and potentially to evolution over time in surgical techniques and perioperative approaches. These results and limitations suggest that the establishment of international defined core outcomes and cross boarder collaboration is essential to advance research in rare disease populations such as EA.

Progress in the treatment of esophageal atresia has been made using an ever-increasing collaborative network. While limited surgical outcome databases have been established for some time, successful large anesthesia outcome networks have only been established more recently in pediatric anesthesia – with the APRICOT and NECTARINE leading examples.^7^^,^^8^ Close collaborations between surgical and pediatric anesthesia networks will permit evaluation of an “esophageal bundle” strategy,^9^ including multidisciplinary perioperative outcomes and long-term follow-up.

## Declaration of competing interest

The authors declare no conflicts of interest.

## References

[1] Sfeir R, Bonnard A, Khen-Dunlop N (2013). Esophageal atresia: data from a national cohort. J Pediatr Surg.

[2] Comella A, Tan Tanny SP, Hutson JM (2021). Esophageal morbidity in patients following repair of esophageal atresia: A systematic review. J Pediatr Surg.

[3] Mirra V, Maglione M, Di Micco LL, Montella S, Santamaria F. (2017). Longitudinal Follow-up of Chronic Pulmonary Manifestations in EsophagealAtresia: A Clinical Algorithm and Review of the Literature. Pediatr Neonatol.

[4] Dellenmark-Blom M, Quitmann J, Dingemann C. (2020). Health-Related Quality of Life in Patients after Repair of Esophageal Atresia: A Review of Current Literature. Eur J Pediatr Surg.

[5] Harrison MS, Goldenberg RL. (2016). Global burden of prematurity. Semin Fetal Neonatal Med.

[6] (1954). The Surgery of Infancy and Childhood: Its Principles and Techniques.Gross RE, Ladd WE. AMA Arch Intern Med..

[7] Disma N, Veyckemans F, Virag K (2021). NECTARINE Group of the European Society of Anaesthesiology Clinical Trial Network; Austria; Belgium; Croatia; Czech Republic; Denmark; Estonia; Finland; France; Germany; Greece; Hungary; Ireland; Italy; Latvia; Lithuania; Luxembourg; Malta; Netherlands; Norway; Poland; Portugal; Romania; Serbia; Slovakia; Slovenia; Spain; Switzerland; Turkey; Ukraine; United Kingdom.Morbidity and mortality after anaesthesia in early life: results of the European prospective multicentre observational study, neonate and children audit of anaesthesia practice in Europe (NECTARINE). Br J Anaesth.

[8] Habre W, Disma N, Virag K (2017). APRICOT Group of the European Society of Anaesthesiology Clinical Trial Network. Incidence of severe critical events in paediatric anaesthesia (APRICOT): a prospective multicentre observational study in 261 hospitals in Europe. Lancet Respir Med.

[9] van den Berg MJ, Johansen M, Disma N (2023). Perioperative anesthetic management and short-term outcome of neonatal repair of esophageal atresia with or without tracheoesophageal fistula in Europe. A sub-analysis of the neonate and children audit of anesthesia practice in Europe (NECTARINE) prospective multicenter observational study. Eur J Anaesthesiol.

